# The Impact of ATRA on Shaping Human Myeloid Cell Responses to Epithelial Cell-Derived Stimuli and on T-Lymphocyte Polarization

**DOI:** 10.1155/2015/579830

**Published:** 2015-04-07

**Authors:** Arunima Chatterjee, Péter Gogolak, Hervé M. Blottière, Éva Rajnavölgyi

**Affiliations:** ^1^Department of Immunology, Medical Faculty, University of Debrecen, Debrecen 4032, Hungary; ^2^INRA, Unité de Virologie et Immunologie Moléculaires, Jouy-en-Josas, France; ^3^AgroParisTech, Jouy-en-Josas, France; ^4^INRA, UMR 1319 Micalis, Jouy-en-Josas, France

## Abstract

Vitamin A plays an essential role in the maintenance of gut homeostasis but its interplay with chemokines has not been explored so far. Using an *in vitro* model system we studied the effects of human colonic epithelial cells (Caco2, HT-29, and HCT116) derived inflammatory stimuli on monocyte-derived dendritic cells and macrophages. Unstimulated Caco2 and HT-29 cells secreted CCL19, CCL21, and CCL22 chemokines, which could attract dendritic cells and macrophages and induced CCR7 receptor up-regulation by retinoic-acid resulting in dendritic cell migration. The chemokines Mk, CXCL16, and CXCL7 were secreted by all the 3 cell lines tested, and upon stimulation by IL-1*β* or TNF-*α* this effect was inhibited by ATRA but had no impact on CXCL1, CXCL8, and CCL20 secretion in response to IL-1*β*. In the presence of ATRA the supernatants of these cells induced CD103 expression on monocyte-derived dendritic cells and when conditioned by ATRA and cocultured with CD4^+^ T-lymphocytes they reduced the proportion of Th17 T-cells. However, in the macrophage-T-cell cocultures the number of these effector T-cells was increased. Thus cytokine-activated colonic epithelial cells trigger the secretion of distinct combinations of chemokines depending on the proinflammatory stimulus and are controlled by retinoic acid, which also governs dendritic cell and macrophage responses.

## 1. Introduction

The adult human intestine is referred to as “physiologically inflamed” due to the presence of enormous number of B- and T-lymphocytes, as well as macrophages (Mf), dendritic cells (DC), eosinophils, and mast cells. If all these immune cells were present in other tissues at such concentrations, they would be regarded as an abnormal chronic inflammatory cell infiltrate [1]. In such a scenario, the main players maintaining gut homeostasis should be those mechanisms that provide tolerogenic signals for specialized myeloid cells with antigen presenting function.

The vitamin A (VitA) metabolite retinoic acid (RA) is a key regulator of the cytokine TGF-*β*, which promotes Treg differentiation [2]. VitA also contributes to the formation of epithelial linings of mucosal surfaces [3], and its multifunctional metabolite RA [4] acts as a critical driver of lymphocyte trafficking to the intestinal mucosa [5]. All-trans retinoic acid (ATRA) induces the expression of the gut homing integrin *α*4*β*7 on myeloid cells and the chemokine receptor CCR9 on T-lymphocytes, while the lack of the *α*v or *β*8 integrin chains in DC impairs Treg functions and Th17 responses* in vivo* [6]. ATRA also modulates Th17 effector T-lymphocyte differentiation in the gut [7]; however, the* in vivo* effects of ATRA in intestinal and extraintestinal compartments result in controversial outcomes presumably due to targeting multiple cell types with diverse functional activities [8]. VitA deficiency has an effect on epithelial cell integrity and the composition of the gut microbiota [9].

A single layer of colonic epithelial cells (CEC) forms the first line of defense against luminal pathogens. It communicates with other immune cells by direct contacts and by secreting an array of cytokines and chemokines. Chemokines represent low-molecular-weight proteins with pleiotropic effects on the recruitment and activation of leukocytes at inflammatory sites [10]. The dominant cell populations in the gut involve CX3CR1^+^ Mf, which directly sense luminal content by their extended membrane protrusions across the epithelium [11], and migratory CD103^+^ DC with tolerogenic potential. Apart from chemokines, colony-stimulating factor (CSF-2/GM-CSF) in the gut is a multifunctional cytokine that has an impact on DC and Mf numbers and can impair the ability of immune cells to produce regulatory factors such as RA and IL-10 and thus may lead to disrupted Treg homeostasis in the large intestine [12]. It also acts as an important regulator of human DC homeostasis by promoting* in vivo* expansion and differentiation from hematopoietic progenitors and monocytes [13]. Under steady state conditions, the low number of gut migratory DC is critically dependent on GM-CSF, but its level is dramatically increased during infection or inflammation and supports the development of DC precursors such as monocytes and inflammatory migratory DC thus modulating the composition of the DC pool [14].

Cytokines have been shown to be the causative factor and outcome of IBD pathogenesis. The major conclusive result has been shown by improvement in the IBD symptoms by blocking TNF-*α*. And a decrease in the IL-1 receptor antagonist in comparison to IL-1 was observed in IBD patients [15]. These data confirm that both TNF-*α* and IL-1*β* are able to trigger inflammatory conditions such as those observed in Crohn's disease (CD) or ulcerative colitis (UC) but the comparison of their effects at molecular and functional levels in context of the human intestinal microenvironment has not been elucidated so far. Despite similarities in the functional and regulatory mechanisms in human and mouse, major differences have been observed in their cytokine secretion [16] and mucus layer organization [17].

Based on these data and to overcome the discrepancies between the human and mouse systems, we designed experiments with human CEC in resting state and in an inflammatory milieu mimicked with TNF-*α* or IL-1*β* stimulation in the presence or absence of ATRA. This was performed by monitoring the levels of secreted chemokines measured at the protein level and by investigating their impact on the phenotype and functional attributes of myeloid cells generated by different growth/differentiation factors. Considering that DC have the potential to instruct T-cells for inflammatory or regulatory directions, our final goal was to identify the impact of stimulated CEC-induced and DC-mediated effects on CD4^+^ effector T-lymphocyte responses. We could detect the secretion of CCL19, CCL21, and CCL22 chemokines by unstimulated CEC, which has not been shown before. We also observed that both IL-1*β* and TNF-*α* were able to trigger the secretion of Midkine (Mk), CXCL16, and CXCL7 by CEC, but their expression could efficiently be downregulated by ATRA. However, the secretion of CXCL1, CXCL8, or CCL20 by IL-1*β*-stimulated CEC was not influenced by ATRA. Our results also revealed that the* in vitro* induced inflammatory milieu created by proinflammatory chemokines was sufficient to increase the migratory potential of DC driven by GM-CSF but not by the other growth factors, and ATRA could further potentiate this effect. Furthermore, the molecular information collected by CEC and transmitted to DC could be translated to T-lymphocytes, which responded to CEC-initiated and DC-mediated stimulation by mounting Th17 responses. All these steps seemed to be under the control of ATRA as the response of CEC to both IL-1*β* and TNF-*α* was higher in the presence of ATRA.

## 2. Materials and Methods

### 2.1. Cell Culture of Caco2 Colon Epithelial Cells

The human colorectal adenocarcinoma cell line Caco2 is from ATCC-number HTB-37 and HT-29 is from ATCC-number HTB-38. The colorectal carcinoma cell line HCT116 was a generous gift from Dr. György Vereb, Department of Biophysics, University of Debrecen. Caco2 and HCT116 cells were cultured in RPMI-1640 medium supplemented with 1% antibiotic-antimycotic solution and 20% fetal bovine serum (GIBCO by Life Technologies, EU) in tissue culture flasks (Nunclon, Rochester, NY) at 37°C in 10% and 5% CO_2_, respectively. HT-29 cells were cultured in RPMI-1640 medium supplemented with 1% antibiotic-antimycotic solution and 10% fetal bovine serum in 5% CO_2_. Cell culture medium was replaced every 2-3 days and the cells were passaged when subconfluent.

### 2.2. Protein Array for Chemokine Analyses

CEC of 70–80% confluency were plated overnight in RPMI supplemented with 10% FCS-followed by stimulation with 10 ng/mL proinflammatory cytokines (IL-1*β* or TNF-*α*) in combination with or without 10 nmol ATRA or left untreated for 1 hour. The cells were washed and replaced with fresh medium for 5 hr, when the supernatants were collected for chemokine analysis performed by a commercially available protein array (Proteome Profiler Arrays—ARY017, R&D Systems, Minneapolis, MN, USA) according to the manufacturer's instructions. Sample controls (transferrin R, gp130, and fibrinogen) included in the array allowed us the detection and quantitation of the secreted chemokines. Considering that ATRA dissolved in DMSO may have toxic effects on resting CEC, which could be further enhanced by activation with IL-1*β* or TNF-*α*, we performed preliminary titration experiments to optimize the cell culture conditions by using 24 h 7AAD-based viability assays performed by FACS analysis. These results indicated 98% viability of Caco2 and HT-29 cells in both the presence and absence of 10 nmol ATRA that was similar to those measured for untreated CEC.

### 2.3. *In Vitro* Cell Migration and the Chemotaxis Assay

Migration of three different groups of monocyte-derived cells, differentiated in GM-CSF+IL-4, GM-CSF, and M-CSF, was tested for cell migration to chemokines and cytokines secreted by Caco2, HT-29, and HCT116 cells. Monocytes (3 × 10^5^) differentiated in the presence of the 3 different growth factors were placed on the upper chamber of a 5-micron Corning transwell plate and the CEC supernatants were added to the lower chamber of the transwell. After 24 h the monocyte-derived cells that migrated to the lower chamber were collected. 10,000 polystyrene beads (15 micron) were added to each sample (Fluka Analytical, Germany) and the number of migrating cells was counted by FACS Calibur (BD Biosciences, Franklin Lakes, NJ, USA). The data were analyzed by the FlowJo software (Tree Star, Ashland, OR, USA).

### 2.4. Peripheral Blood Monocyte-Derived Cells

Leukocyte enriched buffy coats were obtained from healthy blood donors drawn at the Regional Blood Center of the Hungarian National Blood Transfusion Service (Debrecen, Hungary) in accordance with the written approval of the Director of the National Blood Transfusion Service and the Regional and Institutional Ethics Committee of the University of Debrecen, Medical and Health Science Center (Hungary). PBMCs were separated by a standard density gradient centrifugation with Ficoll-Paque Plus (Amersham Biosciences, Uppsala, Sweden). Monocytes were purified from PBMCs by positive selection using immunomagnetic cell separation with anti-CD14 microbeads, according to the manufacturer's instruction (Miltenyi Biotec, Bergisch Gladbach, Germany). After separation on a VarioMACS magnet, 96–99% of the cells were CD14^+^ monocytes, as measured by flow cytometry. Monocytes were divided and cultured in 12-well tissue culture plates at a density of 2 × 10^6^ cells/mL in 10% RPMI medium supplemented with four different growth factors: 80 ng/ml GM-CSF (Gentaur Molecular Products, Brussels, Belgium), 100 ng/mL IL-4 (PeproTech EC, London, UK), and M-CSF 50 ng/mL (MACS, Miltenyi Biotec, Germany).

### 2.5. Peripheral Blood Lymphocytes and CD4^+^ T-Cells

Autologous naive T-cells were separated from human blood mononuclear cells using the naive CD4^+^ T-cell isolation kit based on negative selection according to the manufacturer's instruction (Miltenyi Biotec).

### 2.6. Phenotypic Characterization of Myeloid Cells by Flow Cytometry

Detection of the cell surface expression of monocyte-derived myeloid cells was performed by flow cytometry using anti-CD1a-PE, anti-CD209-PE, anti-CD14-PE, anti-CD83-PE, anti-CD103-PE, anti-CX3CR1-PE, and anti-CCR7-PE (Beckman Coulter, Hialeah, FL, USA). The growth factor receptors were characterized by anti-GM-CSFR*α*-PE and anti-M-CSF R/CD115-PE (R&D Systems, USA) and isotype-matched control antibodies (BD PharMingen, San Diego, CA, USA). Fluorescence intensities were measured by FACS Calibur (BD Biosciences, Franklin Lakes, NJ, USA), and data were analyzed by the FlowJo software (Tree Star, Ashland, OR, USA). The human chemokines Mk, CXCL7, CCL20, and CXCL16 were ordered from PeproTech, UK; CXCL8 and CXCL1 are from Miltenyi Biotec.

### 2.7. IL-17 and IFN*γ* ELISPOT Assays

The monocyte-derived cells were cultured in GM-CSF+IL-4, GM-CSF, and M-CSF for 3 days along with the supernatant of unstimulated or cytokine activated Caco2 cells at 2 × 10^5^ cells/well density. The cells were washed to remove all growth factors and supernatants and were cocultured with naïve autologous CD4^+^ T cells (10^6^cells/well) in 10% RPMI medium for 2 days at 37°C in a humidified atmosphere containing 5% CO_2_. PHA and Con A activated T cells were used as positive controls. Negative controls involved CD4^+^ T-cells and untreated monocyte-derived cells cocultured with CD4^+^ T-cell. Detection of cytokine-secreting T cells was performed by the avidin-HRP system (NatuTec GmbH, Germany). Plates were analyzed by an ImmunoScan plate reader (CTL, Shaker Heights, OH, USA).

### 2.8. Statistical Analysis

Statistical analysis was performed by one-way analysis of variance (ANOVA) for multiple comparisons. Results are expressed as mean ± SD. Two group differences were analyzed by Student's* t*-test.* P* value (two-tailed) less than 0.05 was considered statistically significant.

## 3. Results

### 3.1. Identification of Chemokines Secreted by Resting and Activated CEC

**Figure 1:**
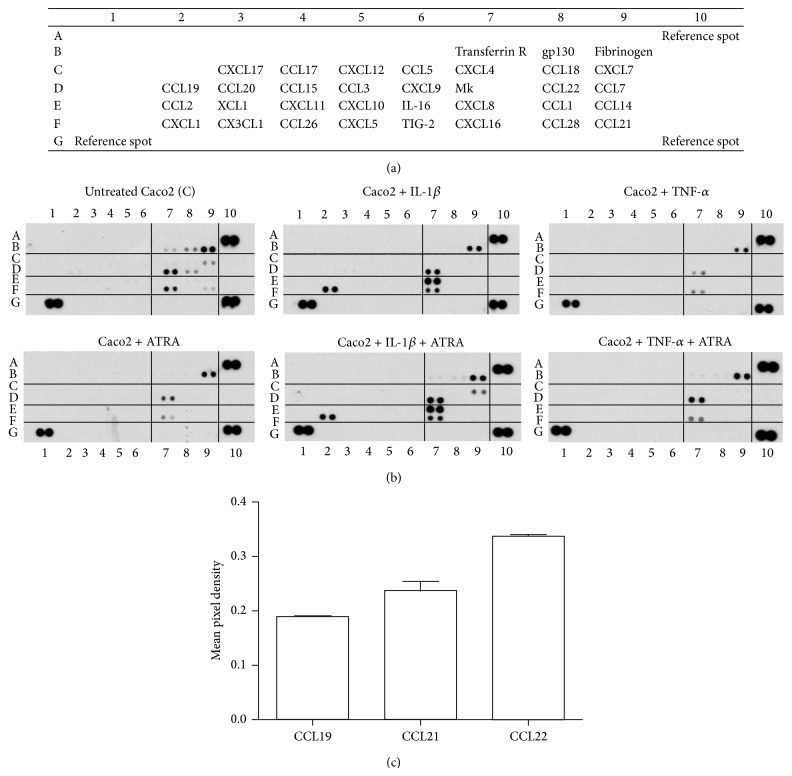
Chemokine secretion of human epithelial cells activated by IL-1*β* or TNF-*α* in the presence or absence of ATRA. Caco2 cells were activated by 10 ng/mL IL-1*β* or TNF-*α* in the presence or absence of 10 nmol ATRA. After 1 hr of activation, the supernatants were removed and the cells were washed and cultured in fresh medium. After 5 hr the supernatants of nontreated and activated Caco2 cells were collected and the relative levels of chemokines were determined by a Proteome Profiler Array used according to the manufacturer's instructions. The relative levels of the Caco2 cell-derived chemokines were determined by calculating mean pixel densities of the individual blots normalized to sample control fibrinogen. The mean ± SD of 4 independent measurements is shown. (a) Localization of the chemokine probes in the membranes related to the positive controls and the reference spots. (b) Representative dot blots showing the relative expression of chemokines produced by untreated Caco2 cells without or with ATRA (upper and lower panels 1) as compared to Caco2 cells stimulated by IL-1*β* (upper panel 2) and IL-1*β* in the presence of ATRA (lower panel 2). Upper and lower panels 3 correspond to Caco2 cells stimulated by TNF-*α* in the absence or presence of ATRA, respectively. (c) Relative expression levels of CCL chemokines produced by unstimulated Caco2 cells.

The single cell monolayer of CEC plays an essential role in the maintenance of gut homeostasis by supporting barrier function and defense against microbes preferentially through the secretion of chemokines [18]. It is also well established that the proinflammatory cytokines IL-1*β* and TNF-*α* act as potent activators of CEC [19]. In this study we applied a high throughput approach for identifying the chemokines secreted by CEC (Caco2, HT-29, and HCT116) in response to IL-1*β* and TNF-*α* by using a commercially available Human Chemokine Array to quantify the relative levels of chemokines released by resting and activated CEC at the protein level. The sample controls provided (transferrin R, gp130, and fibrinogen) were used for calculating mean pixel densities of the respective dot blots. The results showed that resting Caco2 cells secrete detectable levels of CCL19, CCL21, and CCL22 constitutively (dots 2D, 9F, and 8D in Figure 1(a), summarized in Figure 1(c)). Similar results were obtained for HT-29 cells, but HCT116 secreted only CCL19 at detectable levels. Comparison of the relative cytokine levels secreted by the Caco2, HT-29, and HCT116 cell lines are summarized in Table 1. Considering that these CCL chemokines are known to attract myeloid cells, they may maintain a population of myeloid cells in the vicinity of CEC to support cellular interactions. It has previously been shown that MDC/CCL22 attracts Th2 cytokine producing cells and its mRNA and protein expression is upregulated against enteroinvasive bacteria, but inhibition of the NF-*κ*B pathway abolished CCL22 expression in response to proinflammatory stimuli [1]. The chemokines CXCL7, CXCL16, and Mk with different functional activities were also constitutively secreted by resting CEC (dot 9C, 7F, and 7D in Figure 1(a)) suggesting their role in the maintenance of epithelial cell homeostasis.

### 3.2. The Effect of ATRA on the Chemokine Secretion by CEC Prestimulated by IL-1*β* or TNF-*α*

**Figure 2:**
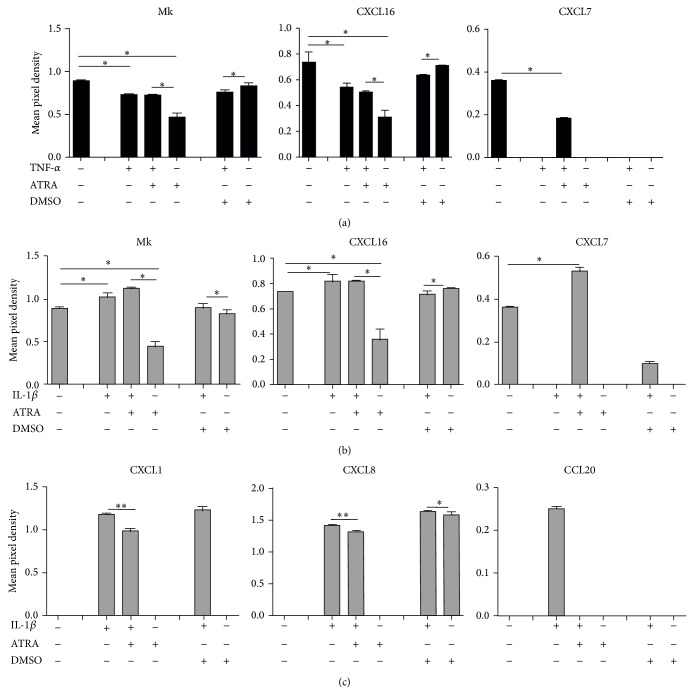
Effects of ATRA on the expression of chemokines in cytokine-stimulated Caco2 cells. Relative expression of chemokines in Caco2 cells, prestimulated by TNF-*α* or IL-1*β* in the presence or absence of ATRA, was determined as described in Figure 1 and was compared to unstimulated cells cultured with or without ATRA. The possible contribution of DMSO used as a solvent control of ATRA was tested in Caco2 cells cultured in the presence of TNF-*α* or IL-1*β* with or without DMSO. (a) Expression of chemokines secreted by Caco2 cells prestimulated by TNF-*α*, in the presence or absence of ATRA. (b) Expression of chemokines secreted by Caco2 cells prestimulated by IL-1*β* in the presence or absence of ATRA. (c) Chemokines induced exclusively by IL-1*β* stimulation in Caco2 cells. ^∗^ *P* < 0.05 , ^∗∗^ *P* < 0.01. Bar diagrams indicate mean ± SD of 4 dot blots which was averaged after densitometry analysis and normalized with the “sample control” provided with the kit.

In our model system, CEC were left untreated or stimulated by IL-1*β* or TNF-*α* in combination with or without ATRA for 6 hr and the cell culture supernatants were subjected to chemokine array analysis by calculating pixel densities using the relevant sample control (Figure 1). When Caco2 cells were activated by TNF-*α* or IL-1*β*, the secretion levels of the chemokines CXCL7, CXCL16, and Mk did not change significantly as compared to unstimulated cells (dots 9C, 7F, and 7D in Figure 1(a), summarized in Figures 2(a) and 2(b)). However, the expression of CXCL16 and Mk was downregulated in the presence of ATRA suggesting that the secretion of these chemokines may contribute to the maintenance of epithelial cell homeostasis. However, under inflammatory conditions they do not mediate positive signals for DC. Remarkably, the secretion of CXCL7 (dot 9C in Figure 1(a), summarized in Figures 2(a) and 2(b)) could be induced only when IL-1*β* or TNF-*α* was combined with ATRA treatment demonstrating the dependence of its secretion on ATRA. DMSO, which is the standard solvent of ATRA, also decreased CXCL7 secretion (Figures 2(a) and 2(b)). Depending on CEC sensitivity to DMSO, certain effects of this solvent have previously been reported, as inhibition of prostaglandin E2 production upon treatment of Caco2 cells with IL-1*β* and attenuation of mRNA levels of IL6, IL-1*α*, and IL-1*β* [20]. These findings might be in line with inhibition of CXCL7 observed in our system. In the IEC-18 cell line IL-1*β* induced increased mRNA levels of MCP-1/CCL2, MIP-1*α*/CCL3, inducible NO synthase, and RANTES/CCL5, which are upregulated in a NF-*κ*B dependent manner [21].

The secretion levels of CCL20 and CXCL1 were not affected by ATRA (dots 3D, 2F, and 7E in Figure 1(a), summarized in Figure 2(c)) and could be induced exclusively by IL-1*β* but not by TNF-*α*. Surprisingly, CXCL8 secretion was upregulated not only by IL-*β* with or without ATRA, but also by DMSO used as a vehicle for ATRA (Figure 2(c)). DMSO induced strain on the actin cytoskeleton and integrins expressed by Caco2 cells was suggested to be transferred to actin-associated molecules like *α*-actinin-1, acting as a scaffold protein and interacted directly with ERK1/2 leading to phosphorylation and increased secretion of CXCL8. The* in vivo* relevance of this effect is underscored by the physical deformation of CEC during peristalsis and villous motility [22]. Along with IL-8/CXCL8 and GRO*α*/CXCL1, Caco2 cells also express MCP-1/CCL2 as a result of IL-1 stimulation, but our chemokine array-based method did not detect CCL2 [23]. These results suggest that the expression of individual chemokines depends on the means of activation and also on ATRA, which can modulate the outcome of chemokine secretion, whereas the group of chemokines not affected by ATRA indicates the complexity of chemokine-mediated regulation in the gut. Similar results were obtained for HT-29 and HCT116 CEC as summarized in Table 1. Even though HCT116 showed a similar overall pattern of chemokine secretion as the other CEC, trace amounts of NF-*κ*B-dependent inflammatory chemokines (CCL2, CXCL2, and CXCL10), not observed in Caco2 and HT29 cells, were detected indicating CEC type-dependent regulation of chemokine secretion [10, 21, 24].

### 3.3. ATRA Regulates the Chemokine-Dependent Migration of Myeloid Cells Generated by Different Hematopoietic Growth/Differentiation Factors

**Figure 3:**
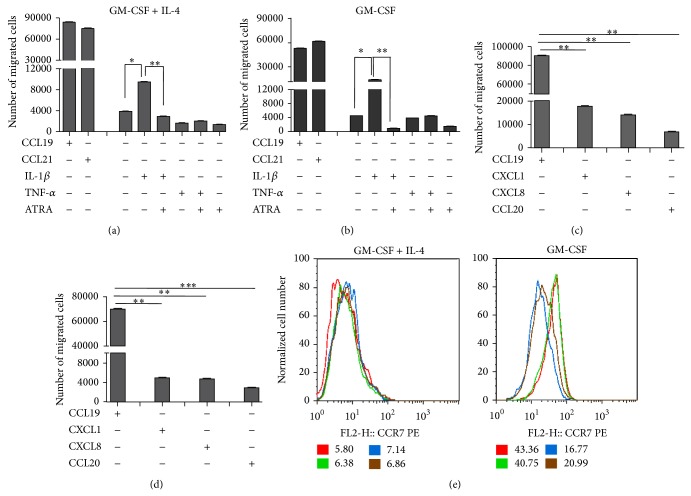
ATRA modifies the chemokine-dependent migration of* in vitro* differentiated myeloid cells. Caco2 cells were activated as described in Figure 1. The migratory potential of the monocyte-derived cells generated by GM-CSF+IL-4 or GM-CSF was tested in transwell chambers. 3 × 10^5^ cells were placed on the upper chamber and the Caco2 cell supernatants on the lower chamber of the transwell plate. After 24 hr, the number of cells, which migrated to the lower chamber in response to the Caco2 cell supernatants, was counted by flow cytometry. The migratory potential of cells generated in the presence of GM-CSF+IL-4 (a) or GM-CSF (b) is shown as compared to the migratory potential of CCL19 and CCL21 chemokines, used as positive controls. To determine the cause of high migration in response to IL-1*β* treated CEC supernatant, the GM-CSF+IL-4 (c) and GM-CSF (d) differentiated monocytes were treated with CXCL1, CXCL8, and CCL20 chemokines which were secreted exclusively by CEC on IL-1*β* treatment. Bar diagrams indicate mean ± SD of 3 independent experiments ^∗^ *P* < 0.05,  ^∗∗^ *P* < 0.01, and  ^∗∗∗^ *P* < 0.001. Another fraction of monocyte-derived cells, differentiated by GM-CSF+IL-4 or GM-CSF, was also stimulated by the supernatant of IL-1*β* pretreated CEC in the presence or absence of ATRA. The cell surface expression of CCR7 in the differentiated monocyte derived cells was measured by flow cytometry (e). Red line depicts untreated monocyte-derived cells, green is for monocyte-derived cells treated by the supernatant of Caco2 cells previously activated by IL-1*β*, blue line is for monocyte-derived cells pretreated with the supernatant of Caco2 cells activated by IL-1*β* and ATRA, and brown line corresponds to cells treated by the supernatant of unstimulated Caco2 cells in combination with ATRA. Results of 3 independent experiments are shown as mean ± SD of MFI.

Based on the results showing the inhibitory effect of ATRA on the secretion of some chemokines, we next sought to assess the chemokine-driven migratory potential of myeloid cells. We set up a transwell system and measured myeloid cell migration* in vitro *by using CCL19 and CCL21 chemokines as positive controls of cell recruitment. Myeloid cells differentiated from primary human monocytes by GM-CSF+IL-4 or GM-CSF to DC exhibited detectable but low migratory potential as compared to cells mobilized by high concentration (200 ng/mL) of CCL19 and CCL21 chemokines (Figures 3(a) and 3(b)), while monocytes cultured in M-CSF gave rise to macrophages with undetectable migratory activity (data not shown). The highest migratory potential could be attributed to cells differentiated in the presence of GM-CSF or GM-CSF+IL-4 and stimulated by the supernatant of Caco2 cells preactivated by IL-1*β*, but this process could be downregulated by ATRA. To analyze whether the supernatant of IL-1*β*-stimulated CEC has a direct effect on the migration of DC differentiated by GM-CSF+IL-4 or GM-CSF, the cells were subjected to direct cell migration assays toward the chemokines exclusively secreted by IL-1*β* used at pretitrated concentrations (CXCL1 (1 ng/mL), CXCL8 (100 ng/mL), and CCL20 (50 ng/mL)). We observed the high migratory capacity of DC differentiated by GM-CSF+IL-4 (Figure 3(c)) and Mf developed by GM-CSF (Figure 3(d)) toward CXCL1 and CXCL8 known to be involved in the chemotaxis and migration of polymorphonuclear leukocytes to inflammatory sites [25, 26] as compared to CCL20. Interestingly, the supernatant of Caco2 cells prestimulated with TNF-*α* had no such effect on cell migration (Figures 3(a) and 3(b)). When the migratory potential of myeloid cells was related to the cell surface expression of CCR7 we found that DC differentiated by GM-CSF in the presence of ATRA-conditioned CEC supernatant exhibited decreased CCR7 expression, while in cells differentiated in GM-CSF+IL-4 it remained unchanged (Figure 3(e)). Similar results were obtained by using the HT-29 and HCT116 cell lines (data not shown). These results suggest that in an inflammatory environment ATRA also modulates the migratory potential of myeloid cells in a cell type-dependent manner.

### 3.4. ATRA Supports the Development of Migratory CD103^+^ Myeloid Cells 

**Figure 4:**
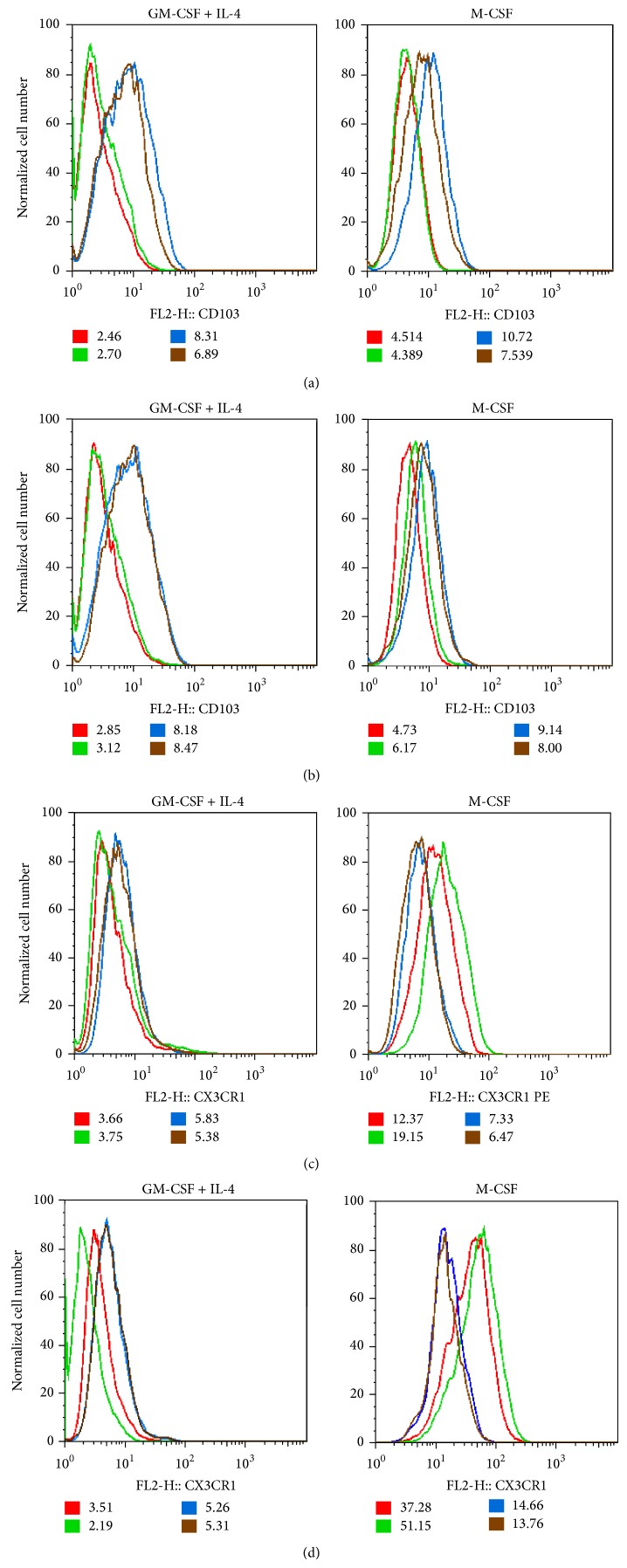
ATRA promotes the expression of CD103^+^ cells in* in vitro* differentiated myeloid cells. Monocytes were differentiated in the presence of GM-CSF+IL-4 or M-CSF, respectively. The differentiated cells were treated by the supernatants of Caco2 cells preactivated by IL-1*β* or TNF-*α* in the presence or absence of ATRA. The phenotype of the myeloid cells was characterized by measuring the cell surface expression of *α*4*β*7/CD103 and CX3CR1 on day 3 of* in vitro *myeloid cell differentiation by flow cytometry. Histograms show the cell surface expression of CD103 in cells differentiated by GM-CSF+IL-4 (or M-CSF) and stimulated by the supernatants of Caco2 cells pretreated by IL-1*β* (a) or TNF-*α* (b) and the cell surface expression of CX3CR1 in cells differentiated by M-CSF (or GM-CSF+IL-4) and stimulated by the supernatants of Caco2 cells prestimulated by IL-1*β* (c) or TNF-*α* (d). MFI of a typical measurement out of 3–5 independent experiments is shown. Red line depicts untreated cells, green is for cells pretreated with the supernatant of Caco2 cells activated by IL-1*β* or TNF-*α*, blue is for myeloid cells pretreated with the supernatant of Caco2 cells activated by IL-1*β* or TNF-*α* in combination with ATRA, and brown corresponds to cells treated by the supernatant of unstimulated Caco2 cells and ATRA.

The dominant DC population of the gut is represented by CD103^+^ migratory cells that express the enzymes required for the metabolism of VitA [4], while the CX3CR1^+^ resident Mf population samples the microenvironment by protruding dendrites [11]. To assess how efficiently we could manipulate the effects of ATRA on CEC, we differentiated blood-derived monocytes with GM-CSF+IL-4 or M-CSF to generate DC and Mf, respectively, followed by the stimulation of cells with the supernatant of activated Caco2 cells. The cell surface expression of CD103^+^ and CX3CR1^+^ measured by FACS analysis revealed that the presence of Caco2 cell supernatants obtained from ATRA+IL-1*β*, ATRA+TNF-*α*, or ATRA-pretreated CEC could increase the expression of the CD103 integrin in cells differentiated by GM-CSF+IL-4 or M-CSF to obtain DC and Mf, respectively (Figures 4(a) and 4(b)). These results also indicated that even in the presence of inflammatory stimuli (supernatant of IL-1*β* or TNF-*α* activated CEC) ATRA was able to promote the development of CD103^+^ myeloid cells. In a similar experimental system, the frequency of CX3CR1^+^ cells generated by GM-CSF+IL-4 and stimulated by cytokine-activated CEC supernatant was also increased in case the cell culture was conditioned by ATRA. In contrast to this finding, the expression of CX3CR1 remained unchanged in cells generated by GM-CSF (data not shown) and was decreased when the ATRA-conditioned CEC supernatant was added to Mf differentiated from monocytes with M-CSF and activated by IL-1*β* or TNF-*α* (Figures 4(c) and 4(d)). Similar results were obtained with HT-29 and HCT116 cells (data not shown). To confirm that the level of ATRA could be reconstituted in CEC after the washing procedure, we also added ATRA directly to DC and detected increased expression of CD103. To rule out the effect of other chemokines in the development of CD103^+^cells, the chemokines secreted by activated CEC were added directly to monocytes at pretitrated concentrations (Mk 10 ng/mL, CXCL16 100 ng/mL, CXCL7 10 ng/mL, CXCL1 1 ng/mL, CXCL8 100 ng/mL, and CCL20 50 ng/mL) along with the differentiating growth factors GM-CSF+IL-4 or M-CSF and the expression of CD103 was measured by FACS. When the* in vitro* generated myeloid cells were treated directly with the different chemokines in the absence of ATRA, no phenotypic changes of DC and Mf could be detected (see Supplementary Figure  4 in Supplementary Material available online at http://dx.doi.org/10.1155/2015/579830), showing their direct dependence on ATRA.

### 3.5. Translation of the Molecular Information Collected by CEC-Stimulated Myeloid Cells to CD4^+^ T-Lymphocytes

**Figure 5:**
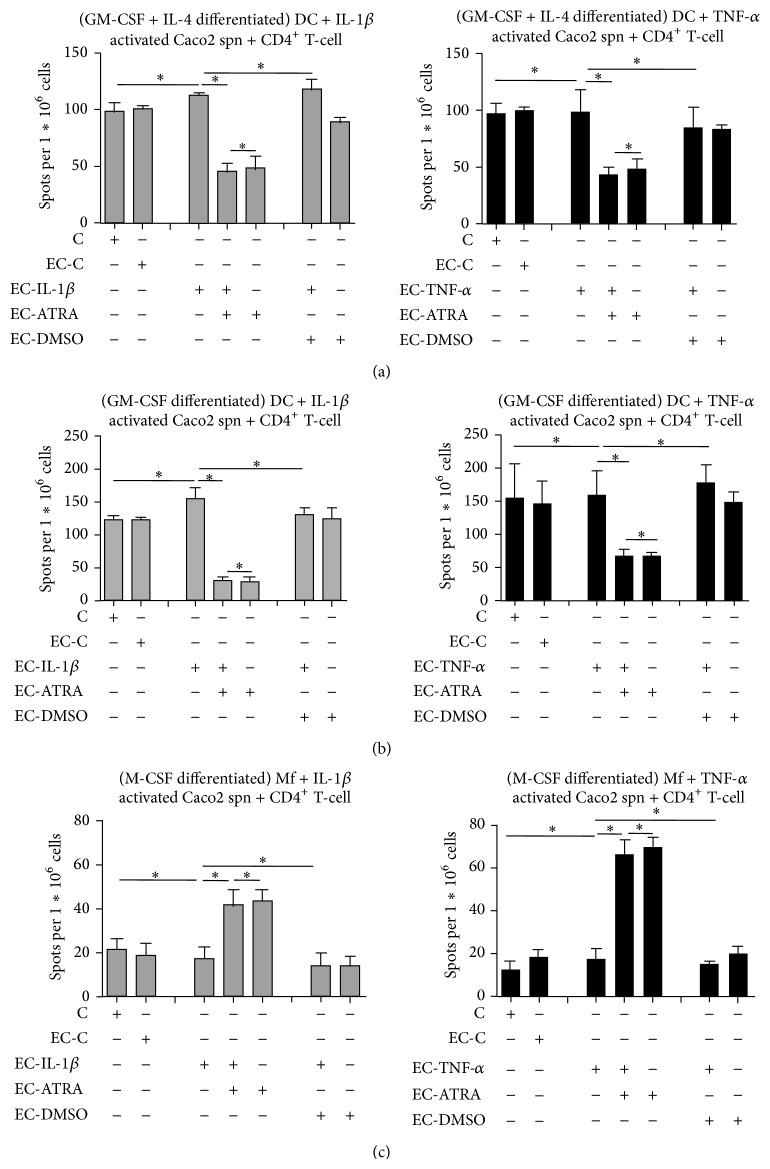
The effect of gut epithelial cell-mediated myeloid cell stimulation on the activation and polarization of T-lymphocytes. To assess the effects of stimulated myeloid cells on effector T-cell polarization, the supernatants of activated Caco2 cells were added to myeloid cells differentiated by GM-CSF+IL-4, GM-CSF, or M-CSF for 3 days and the washed cells were cocultured in fresh medium with autologous CD4^+^ T-lymphocytes for another 2 days. The activation and polarization of CD4^+^ T-cells c-incubated with myeloid cells generated by GM-CSF+IL-4 (a), GM-CSF (b), or M-CSF (c) was detected by the IL-17 ELISPOT assay. In the figure legends, C stands for unstimulated myeloid cells coincubated with CD4^+^ T cells, EC-C for monocyte-derived cells “educated” by resting Caco2 cell supernatant followed by coincubation with CD4^+^ T cells, and EC-IL-1*β*, EC-TNF-*α*, EC-ATRA, and EC-DMSO correspond to cultures containing monocyte-derived cells “educated” with the supernatants of Caco2 cells pretreated with IL-1*β* or TNF-*α* in the presence or absence of ATRA, or with DMSO used as solvent control, followed by coincubation with CD4^+^ T cells. ^∗^ *P* < 0.05. Mean ± SD of the number of IL-17 secreting cells measured in 4 independent experiments is shown.

Considering the sensitivity of myeloid cells to proinflammatory signals provided by activated CEC and the modulatory effects of ATRA, we set out to test whether DC and Mf as antigen presenting cells could activate and polarize T-lymphocytes. To test this scenario, myeloid cells differentiated by GM-CSF+IL-4, GM-CSF, and M-CSF, respectively, were activated by supernatants of cytokine-activated Caco2 cells followed by coculturing them with autologous CD4^+^ T-lymphocytes, and the number of IL-17 and IFN*γ* cytokine producing T-cells was detected by ELISPOT assays. We found that CD4^+^ T cells cocultured with myeloid cells differentiated from monocytes to DC with GM-CSF+IL-4 (Figure 5(a)) or GM-CSF (Figure 5(b)) and “educated” by the supernatants of activated Caco2 cells in combination with ATRA resulted in significant suppression of IL-17 producing cell numbers. In contrast, monocyte-derived cells generated by M-CSF pretreated with Caco2 cell supernatant (Figure 5(c)) and subsequently co-cultured with CD4^+^ T-cells, the number of IL-17 cytokine secreting cells was increased significantly indicating that DC and Mf exhibit different T-cell polarizing activities. Although slight differences could be observed in the magnitude of T-cell responses provoked by CEC supernatants activated by IL-1*β* or TNF-*α*, the T-lymphocyte responses were polarized to the Th17 direction in both cases independent of the proinflammatory cytokine used for CEC stimulation underpinning the role of DC-mediated inflammatory signals in driving CD4^+^ T-lymphocyte responses. Under similar culture conditions IFN*γ*-secreting cells could not be detected in the CD4^+^ T-cell population. Similar “education” of myeloid cells by ATRA-conditioning was observed also for HT-29 and HCT116 cell lines (data not shown). Thus, ATRA is able to exert different effects on monocyte-derived myeloid cells differentiated upon coculturing with CD4^+^ T cells. This may indicate a broad range of RA-mediated effects involved in shaping the gut microenvironment.

## 4. Discussion

The cytokines secreted at increased levels in patients with IBD have been identified as TNF-*α* and IL-1*β*, but the complete spectrum of chemokines and chemokine receptors involved in these regulatory networks has not been analyzed in detail. We designed an* in vitro* experimental system to study the effects and the interplay of cytokines, chemokines, and RA in resting CEC and under inflammatory conditions for identifying the possible outcomes of myeloid cell-induced T-cell collaboration (Supplementary Figure  1). We observed that unstimulated CEC secrete CCL chemokines with the potential to attract DC and Mf thus ensuring continuous contact with CEC to support LP homeostasis [27, 28]. It has previously been observed that TNF-*α* and IL-1*β* do not induce the secretion of CCL22 in monocytes, macrophages, and B cells from human peripheral blood* in vitro*, but prolonged (12 hr) treatment could induce production of MDC/CCL22 protein by cultured human intestinal epithelial cells [1]. The detailed analysis of chemokine expression induced by the supernatants of CEC preactivated by IL-1*β* or TNF-*α* demonstrated that (1) the secretion of the CCL20, CXCL1, and CXCL8 chemokines could be induced only by IL-1*β* and was not affected by ATRA, (2) constitutive expression of the chemokines Mk, CXCL16, and CXCL7 was not modified by the supernatant of activated CEC, (3) ATRA downregulated the expression of Mk and CXCL16, and (4) the secretion of CXCL7 could be induced by both IL-1*β* and TNF-*α* in the presence of ATRA. Consistent with previous results, we also observed in our* in vitro* model that CXCL8 and CXCL1 are secreted upon activation of CEC by IL-1*β* but not by TNF-*α* showing that IL-1 family cytokines may exert dichotomous or opposing effects in maintaining gut homeostasis or inducing intestinal inflammation [29]. When Caco2 cells were treated with TNF-*α* in combination with* Clostridium difficile* toxin A, TNF-*α* itself did not influence the secretion of CXCL8 [10].  The chemokine CCL20 exhibited unique features as it could be induced exclusively by the supernatant of IL-1*β* stimulated CEC that could completely be inhibited by ATRA. This chemokine is highly specific for its receptor CCR6, that is, expressed by intestinal CEC and in human lymphoid tissues [30–32] and its expression is associated with IBD [33, 34]. The importance of the CCR6-CCL20 axis was also verified in CCR6 double negative mice showing decreased intestinal M-cell numbers and low IgA secretion upon rotavirus infection [35, 36]. Mk acts as a multifunctional cytokine and growth factor with bactericidal and fungicidal activity. Upregulation of Mk expression was also detected in the rat large intestine during DSS-induced colitis and was shown to activate CD4^+^ T cells [37] leading to enhanced mucosal restitution during the repair process of colitis [38]. Ligation of the CXCR6 receptor by its CXCL16 ligand results in the activation of the MAP-kinase pathway observed in patients with Crohn's disease and was associated with clinical benefits and rapid ulcer healing [39]. When CEC were stimulated by IL-1*β* or TNF-*α*, the secretion levels of Mk, CXCL16, and CXCL7 remained constant while the physiological concentrations of ATRA could decrease the secretion of these chemokines significantly. CXCL7 was also shown to promote neutrophil adhesion and transmigration [40].

GM-CSF is a critical factor for DC development and expansion* in vivo;* it induces DC differentiation from monocytes and its absence can reduce the number of migratory DC [41, 42].

Based on this information and to further characterize the cell types involved, we generated myeloid cells with characteristic phenotypic properties by differentiating them in the presence of various growth factors (Table 2). We observed that the myeloid cells differentiated with ATRA exerted cell type-specific modulatory effects on their phenotype shown by the increased expression of CD14, GM-CSF, and M-CSF receptors, while decreasing CCR7 expression and inhibiting memory T-cell migration to secondary lymphoid organs [43] (Supplementary Figure  2). The role of ATRA in this process was confirmed by* in vitro* migration assays detecting moderate cell migration towards the supernatant of activated CEC containing a selected set of chemokines induced by activated CEC. ATRA inhibited the migration of myeloid cells differentiated in the presence of various growth factors even when they were stimulated by the supernatant of IL-1*β*-activated Caco2 cells. These results suggest that ATRA is a potent regulator of the tolerogenic microenvironment in the gut acting at least partially via modulating chemokine responses. Despite slight differences in the levels of CEC-derived chemokine secretion, the overall effects on modulating the myeloid cell phenotype, the migratory potential, and the DC- and Mf-mediated polarization of CD4^+^ T-lymphocytes were comparable for the tested CEC that involved the Caco2, HT-29, and HCT116 cell lines suggesting a common regulatory mechanism.

Inhibited expression of the LPS-binding receptor CD14 (Supplementary Figure  2(a)) was shown to be associated with increased susceptibility to gastroenteritis and UC [44] and the expression of GM-CSF receptor was shown to be higher in healthy individuals than in UC or CD patients [45]. Microbial signals sensed by Mf in the colon are dependent on GM-CSF and result in increased IL-1*β* secretion. In the absence of microbes, decreased IL-1*β* secretion of mice led to low GM-CSF levels in the gut, while DC and Mf support the generation of Tregs by producing RA and IL-10 in the presence of TGF-*β*. The production of these regulators was drastically decreased in the absence of GM-CSF, as shown in *Csf*2−/−  mice [12]. In accordance with these findings we also observed the secretion of IL-1*β* when monocyte-derived cells generated in GM-CSF were treated with the supernatant of Caco2 cells conditioned by ATRA (Supplementary Figure  3). In this setting ATRA also decreased GM-CSF receptor expression (Supplementary Figure  2(b)) which could contribute to keep the overall concentration of IL-1*β* in the gut environment to controllable limits. ATRA is also responsible for the homeostatic regulation of CD11b^+^CD103^+^ DC [46] and under inflammatory conditions its production is increased to keep the local environment under check. When the chemokines were added directly to DC to test their effects on CD103 expression, DC could not acquire CD103 surface expression in the absence of ATRA (Supplementary Figure  4). Based on these results, we suggest that human monocyte-derived CD103^+^ cells, induced by GM-CSF+IL-4 or GM-CSF together with appropriate activation signals, that is, the supernatant of activated CEC, are able to support the acquisition of the gut phenotype of human DC. To confirm this finding, we also performed experiments with blood-derived CD1c^+^ DC and obtained a similar outcome (unpublished results) indicating that in the human system both cell types can acquire the capability to support CD103^+^ myeloid cell development. Differentiation of the CX3CR1^+^ population in the presence of GM-CSF+IL-4 was also promoted by ATRA but it was inhibited in Mf. These results are in line with previous results showing that CX3CR1^+^ cells are inefficient in synthesizing ATRA and exhibit poor T-cell stimulatory capacity* in vitro* and* in vivo* when injected into intestinal lymphatics [47]. In contrast, CD103^+^cells are able to migrate and can trigger adaptive immune responses by expressing gut homing receptors on T-cells [47].

The expression of M-CSFR (Supplementary Figure  2(b)) was shown to be enhanced by IL-1*β* stimulated CEC supernatant and the number of Th17 cells was increased when CD4^+^ T cells were coincubated with Mf educated by the supernatant of CEC preactivated by IL-1*β* in the presence of physiological concentration of ATRA. The decreased number of Treg cell as compared to Th17 cells has been indicated in Crohn's patients and the number of Tregs was influenced by RA and IL-10 in the gut environment [10]. Bone marrow-derived DC could be “educated” by direct contact with murine epithelial MODE-K cells and the development of surface expression of CD103^+^ was observed, which is involved directly in the development of Treg differentiation [48]. In our* in vitro* model with human CEC (Caco2, HT-29, and HCT116) and monocyte-derived DC, the development of Tregs was not observed. However, we were able to demonstrate the inhibition of Th17 cell numbers when autologous T-cells were cocultured with “ATRA educated” DC. We also observed the enhanced secretion of IL-10 by CD4^+^T-cells after coculturing them with ATRA-conditioned DC and Mf (Supplementary Figure  5) indicating suboptimal conditions for Treg differentiation. It has also been shown that Th17 cells can exhibit both anti- and proinflammatory properties depending on the cytokine signals received [49]. These results altogether indicated that the detailed characterization of the gut milieu under different conditions is of utmost importance for understanding the complexity of regulation leading to the maintenance or loss of gut homeostasis.

## 5. Conclusion

The proinflammatory cytokines IL-1*β* and TNF-*α*, associated with inflammatory bowel diseases, exert different effects on gut epithelial cells monitored by the secretion of different combinations of chemokines in CEC. The metabolite RA, produced by both epithelial and myeloid cells, has the potential to downmodulate some, but not all chemokine responses to support a tolerogenic microenvironment in the gut environment. Myeloid cells differentiated by GM-CSF+IL-4 or GM-CSF (DC) and M-CSF (Mf) respond differently to activated epithelial cell-mediated stimuli and to RA. The signals provided by activated epithelial cells for DC and Mf can be translated to T-lymphocytes, but the number of Th17 producing cells is modulated differentially in DC and Mf indicating the complementary role of these myeloid cell types in regulating adaptive effector T-cell polarization.

## Supplementary Material

Supplementary Figures involve the outline of the experimental setting, shows the phenotype of the cells generated in the presence of different growth factors and the effects of ATRA on CD103+ myeloid cells as well as the effect of GM-CSF in the autocrine secretion of IL-1β and the homeostatic secretion of IL-10 by CD4+ T-lymphocytes.

## Figures and Tables

**Table 1:** Relative expression of chemokines secreted by colon epithelial cell lines. Caco2, HT-29, and HCT116 cells were treated with IL-1*β* or TNF-*α* in the presence or absence of ATRA for 60 min. After removing the cell culture supernatant the cells were washed and replaced with fresh medium and the supernatants were collected after 5 hours and used for the Chemokine Array analysis according to the manufacturer's instruction to determine the variety of chemokines secreted by these cell lines. The values indicate the results of the densitometric analysis of dot blots and are normalized to the relevant sample control according to the manufacturer's instructions. For simplicity the value of 1 obtained after dividing with the sample control is referred to as 100.

		CCL19	CCL21	CCL22	Mk	CXCL16	CXCL7	CXCL1	CXCL8	CCL20
C	Caco2	30	30	30	90	70	40	—	—	—
	HT-29	40	30	40	80	90	60	—	—	—
	HCT-116	150	—	—	170	170	—	—	120	—

IL-1*β*	Caco2	—	—	—	100	80	—	120	140	30
	HT-29	—	—	—	80	90	—	100	90	30
	HCT-116	—	—	—	150	160	100	100	100	100

IL-1*β* + ATRA	Caco2	—	—	—	110	80	50	100	130	—
	HT-29	—	—	—	100	80	40	100	90	—
	HCT-116	—	—	—	160	140	90	110	110	—

TNF-*α*	Caco2	—	—	—	90	60	—	—	—	—
	HT-29	—	—	—	80	60	—	—	—	—
	HCT-116	—	—	—	150	150	—	10	10	—

TNF-*α* + ATRA	Caco2	—	—	—	90	60	—	—	—	—
	HT-29	—	—	—	90	80	20	—	—	—
	HCT-116	—	—	—	150	130	20	10	—	—

ATRA	Caco2	—	—	—	50	30	—	—	—	—
	HT-29	—	—	—	60	20	—	—	—	—
	HCT-116	40	20	30	80	80	—	—	—	—

**Table 2:** Expression of CD1a, DC-SIGN, and CD14 cell surface molecules on monocyte-derived cells generated in the presence of the colony stimulating factors GM-CSF+IL-4, GM-CSF, or M-CSF. Peripheral blood-derived monocytes were differentiated in the presence of GM-CSF+IL-4, GM-CSF, or M-CSF in RPMI + 10% FCS for 3 days and the expression of the cell surface markers CD1a, DC-SIGN/CD209, and CD14 was monitored by flow cytometry using fluorescent labeled specific antibodies. The expression levels of CD1a, DC-SIGN, and CD14 are indicated as mean fluorescence intensity (MFI).

	CD1a	DC SIGN	CD14
GMCSF+IL-4	200.88	32.6	75.63
GMCSF	66.31	10.5	183.29
MCSF	6.01	4.3	369.47

## References

[1] Berin M. C., Dwinell M. B., Eckmann L., Kagnoff M. F. (2001). Production of MDC/CCL22 by human intestinal epithelial cells. *The American Journal of Physiology—Gastrointestinal and Liver Physiology*.

[2] Mucida D., Park Y., Kim G. (2007). Reciprocal TH17 and regulatory T cell differentiation mediated by retinoic acid. *Science*.

[3] Mark M., Ghyselinck N. B., Chambon P. (2006). Function of retinoid nuclear receptors: lessons from genetic and pharmacological dissections of the retinoic acid signaling pathway during mouse embryogenesis. *Annual Review of Pharmacology and Toxicology*.

[4] Szatmari I., Pap A., Rühl R. (2006). PPAR*γ* controls CD1d expression by turning on retinoic acid synthesis in developing human dendritic cells. *Journal of Experimental Medicine*.

[5] Iwata M., Eshima Y., Kagechika H. (2003). Retinoic acids exert direct effects on T cells to suppress T_h_1 development and enhance T_h_2 development via retinoic acid receptors. *International Immunology*.

[6] Wurbel M.-A., McIntire M. G., Dwyer P., Fiebiger E. (2011). CCL25/CCR9 interactions regulate large intestinal inflammation in a murine model of acute colitis. *PLoS ONE*.

[7] Villablanca E. J., Cassani B., von Andrian U. H., Mora J. R. (2011). Blocking lymphocyte localization to the gastrointestinal mucosa as a therapeutic strategy for inflammatory bowel diseases. *Gastroenterology*.

[8] McDonald K. G., Leach M. R., Brooke K. W. M. (2012). Epithelial expression of the cytosolic retinoid chaperone cellular retinol binding protein II is essential for in vivo imprinting of local gut dendritic cells by lumenal retinoids. *American Journal of Pathology*.

[9] Cha H.-R., Chang S.-Y., Chang J.-H. (2010). Downregulation of Th17 cells in the small intestine by disruption of gut flora in the absence of retinoic acid. *Journal of Immunology*.

[10] Kim J. M., Kim J. S., Jung H. C., Oh Y.-K., Song I. S., Kim C. Y. (2002). Differential expression and polarized secretion of CXC and CC chemokines by human intestinal epithelial cancer cell lines in response to Clostridium difficile toxin A. *Microbiology and Immunology*.

[11] Chieppa M., Rescigno M., Huang A. Y. C., Germain R. N. (2006). Dynamic imaging of dendritic cell extension into the small bowel lumen in response to epithelial cell TLR engagement. *Journal of Experimental Medicine*.

[12] Mortha A., Chudnovskiy A., Hashimoto D. (2014). Microbiota-dependent crosstalk between macrophages and ILC3 promotes intestinal homeostasis. *Science*.

[13] Sallusto F., Nicolò C., de Maria R., Corinti S., Testi R. (1996). Ceramide inhibits antigen uptake and presentation by dendritic cells. *Journal of Experimental Medicine*.

[14] Varol C., Zigmond E., Jung S. (2010). Securing the immune tightrope: mononuclear phagocytes in the intestinal lamina propria. *Nature Reviews Immunology*.

[15] Neurath M. F. (2014). Cytokines in inflammatory bowel disease. *Nature Reviews Immunology*.

[16] Jarry A., Bossard C., Bou-Hanna C. (2008). Mucosal IL-10 and TGF-*β* play crucial roles in preventing LPS-driven, IFN-*γ*-mediated epithelial damage in human colon explants. *Journal of Clinical Investigation*.

[17] Te Velde A. A., Verstege M. I., Hommes D. W. (2006). Critical appraisal of the current practice in murine TNBS-induced colitis. *Inflammatory Bowel Diseases*.

[18] Shang L., Thirunarayanan N., Viejo-Borbolla A. (2009). Expression of the chemokine binding protein M3 promotes marked changes in the accumulation of specific leukocytes subsets within the intestine. *Gastroenterology*.

[19] Lacruz-Guzmán D., Torres-Moreno D., Pedrero F. (2013). Influence of polymorphisms and TNF and IL1*β* serum concentration on the infliximab response in Crohn's disease and ulcerative colitis. *European Journal of Clinical Pharmacology*.

[20] Hollebeeck S., Raas T., Piront N. (2011). Dimethyl sulfoxide (DMSO) attenuates the inflammatory response in the in vitro intestinal Caco-2 cell model. *Toxicology Letters*.

[21] Yan S. R., Joseph R. R., Wang J., Stadnyk A. W. (2006). Differential pattern of inflammatory molecule regulation in intestinal epithelial cells stimulated with IL-1. *Journal of Immunology*.

[22] Craig D. H., Zhang J., Basson M. D. (2007). Cytoskeletal signaling by way of *α*-actinin-1 mediates ERK1/2 activation by repetitive deformation in human Caco2 intestinal epithelial cells. *American Journal of Surgery*.

[23] Warhurst A. C., Hopkins S. J., Warhurst G. (1998). Interferon *γ* induces differential upregulation of *α* and *β* chemokine secretion in colonic epithelial cell lines. *Gut*.

[24] Torres J., Danese S., Colombel J.-F. (2013). New therapeutic avenues in ulcerative colitis: thinking out of the box. *Gut*.

[25] Kaur M., Singh D. (2013). Neutrophil chemotaxis caused by chronic obstructive pulmonary disease alveolar macrophages: the role of CXCL8 and the receptors CXCR1/CXCR2. *Journal of Pharmacology and Experimental Therapeutics*.

[26] Cremel M., Berlier W., Hamzeh H. (2005). Characterization of CCL20 secretion by human epithelial vaginal cells: involvement in Langerhans cell precursor attraction. *Journal of Leukocyte Biology*.

[27] Cravens P. D., Lipsky P. E. (2002). Dendritic cells, chemokine receptors and autoimmune inflammatory diseases. *Immunology and Cell Biology*.

[28] Weber M., Hauschild R., Schwarz J. (2013). Interstitial dendritic cell guidance by haptotactic chemokine gradients. *Science*.

[29] Lopetuso L. R., Chowdhry S., Pizarro T. T. (2013). Opposing functions of classic and novel IL-1 family members in gut health and disease. *Frontiers in Immunology*.

[30] Varona R., Zaballos A., Gutiérrez J. (1998). Molecular cloning, functional characterization and mRNA expression analysis of the murine chemokine receptor CCR6 and its specific ligand MIP-3*α*. *FEBS Letters*.

[31] Liao F., Alderson R., Su J., Ullrich S. J., Kreider B. L., Farber J. M. (1997). STRL22 is a receptor for the CC chemokine MIP-3*α*. *Biochemical and Biophysical Research Communications*.

[32] Baba M., Imai T., Nishimura M. (1997). Identification of CCR6, the specific receptor for a novel lymphocyte- directed CC chemokine LARC. *The Journal of Biological Chemistry*.

[33] Rossi D. L., Vicari A. P., Franz-Bacon K., McClanahan T. K., Zlotnik A. (1997). Identification through bioinformatics of two new macrophage proinflammatory human chemokines: MIP-3*α* and MIP-3*β*. *Journal of Immunology*.

[34] Barrett J. C., Hansoul S., Nicolae D. L. (2008). Genome-wide association defines more than 30 distinct susceptibility loci for Crohn's disease. *Nature Genetics*.

[35] Cook D. N., Prosser D. M., Forster R. (2000). CCR6 mediates dendritic cell localization, lymphocyte homeostasis, and immune responses in mucosal tissue. *Immunity*.

[36] Lügering A., Floer M., Westphal S. (2005). Absence of CCR6 inhibits CD4^+^ regulatory T-cell development and M-cell formation inside Peyer's patches. *The American Journal of Pathology*.

[37] Kerzerho J., Schneider A., Favry E., Castelli F. A., Maillère B. (2013). The signal peptide of the tumor-shared antigen midkine hosts CD4^+^ T cell epitopes. *The Journal of Biological Chemistry*.

[38] Yuki T., Ishihara S., Rumi M. A. K. (2006). Increased expression of midkine in the rat colon during healing of experimental colitis. *The American Journal of Physiology—Gastrointestinal and Liver Physiology*.

[39] Diegelmann J., Seiderer J., Niess J.-H. (2010). Expression and regulation of the chemokine CXCL16 in Crohn's disease and models of intestinal inflammation. *Inflammatory Bowel Diseases*.

[40] Schenk B. I., Petersen F., Flad H.-D., Brandt E. (2002). Platelet-derived chemokines CXC chemokine ligand (CXCL)7, connective tissue-activating peptide III, and CXCL4 differentially affect and cross-regulate neutrophil adhesion and transendothelial migration. *Journal of Immunology*.

[41] Bogunovic M., Ginhoux F., Helft J. (2009). Origin of the lamina propria dendritic cell network. *Immunity*.

[42] van de Laar L., Coffer P. J., Woltman A. M. (2012). Regulation of dendritic cell development by GM-CSF: molecular control and implications for immune homeostasis and therapy. *Blood*.

[43] Shields J. D., Fleury M. E., Yong C., Tomei A. A., Randolph G. J., Swartz M. A. (2007). Autologous chemotaxis as a mechanism of tumor cell homing to lymphatics via interstitial flow and autocrine CCR7 signaling. *Cancer Cell*.

[44] Mohamed J. A., Dupont H. L., Flores J. (2011). Single nucleotide polymorphisms in the promoter of the gene encoding the lipopolysaccharide receptor CD14 are associated with bacterial diarrhea in US and Canadian travelers to Mexico. *Clinical Infectious Diseases*.

[45] Goldstein J. I., Kominsky D. J., Jacobson N. (2011). Defective leukocyte GM-CSF receptor (CD116) expression and function in inflammatory bowel disease. *Gastroenterology*.

[46] Greter M., Helft J., Chow A. (2012). GM-CSF controls nonlymphoid tissue dendritic cell homeostasis but is dispensable for the differentiation of inflammatory dendritic cells. *Immunity*.

[47] Schulz O., Jaensson E., Persson E. K. (2009). Intestinal CD103+, but not CX3CR1+, antigen sampling cells migrate in lymph and serve classical dendritic cell functions. *Journal of Experimental Medicine*.

[48] Iliev I. D., Mileti E., Matteoli G., Chieppa M., Rescigno M. (2009). Intestinal epithelial cells promote colitis-protective regulatory T-cell differentiation through dendritic cell conditioning. *Mucosal Immunology*.

[49] Ramesh R., Kozhaya L., McKevitt K. (2014). Pro-inflammatory human Th17 cells selectively express P-glycoprotein and are refractory to glucocorticoids. *Journal of Experimental Medicine*.

