# Supplementary material for: The Impact of ATRA on Shaping Human Myeloid Cell Responses to Epithelial Cell-Derived Stimuli and on T-Lymphocyte Polarization
Source: Mediators Inflamm. 2015 Apr 7;2015:579830. doi: 10.1155/2015/579830 (PMC4405019; doi:10.1155/2015/579830)
Supplement: Supplementary file 1 — Supplementary Figures involve the outline of the experimental setting, shows the phenotype of the cells generated in the presence of different growth factors and the effects of ATRA on CD103+ myeloid cells as well as the effect of GM-CSF in the autocrine secretion of IL-1β and the homeostatic secretion of IL-10 by CD4+ T-lymphocytes. [file 579830.f1.pdf]

Supplementary Figure 1

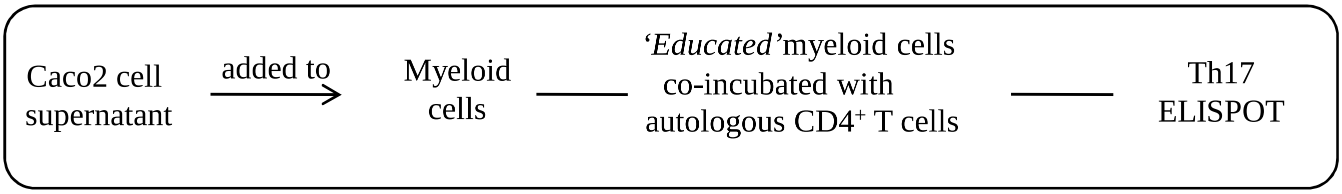

Supplementary Figure 2

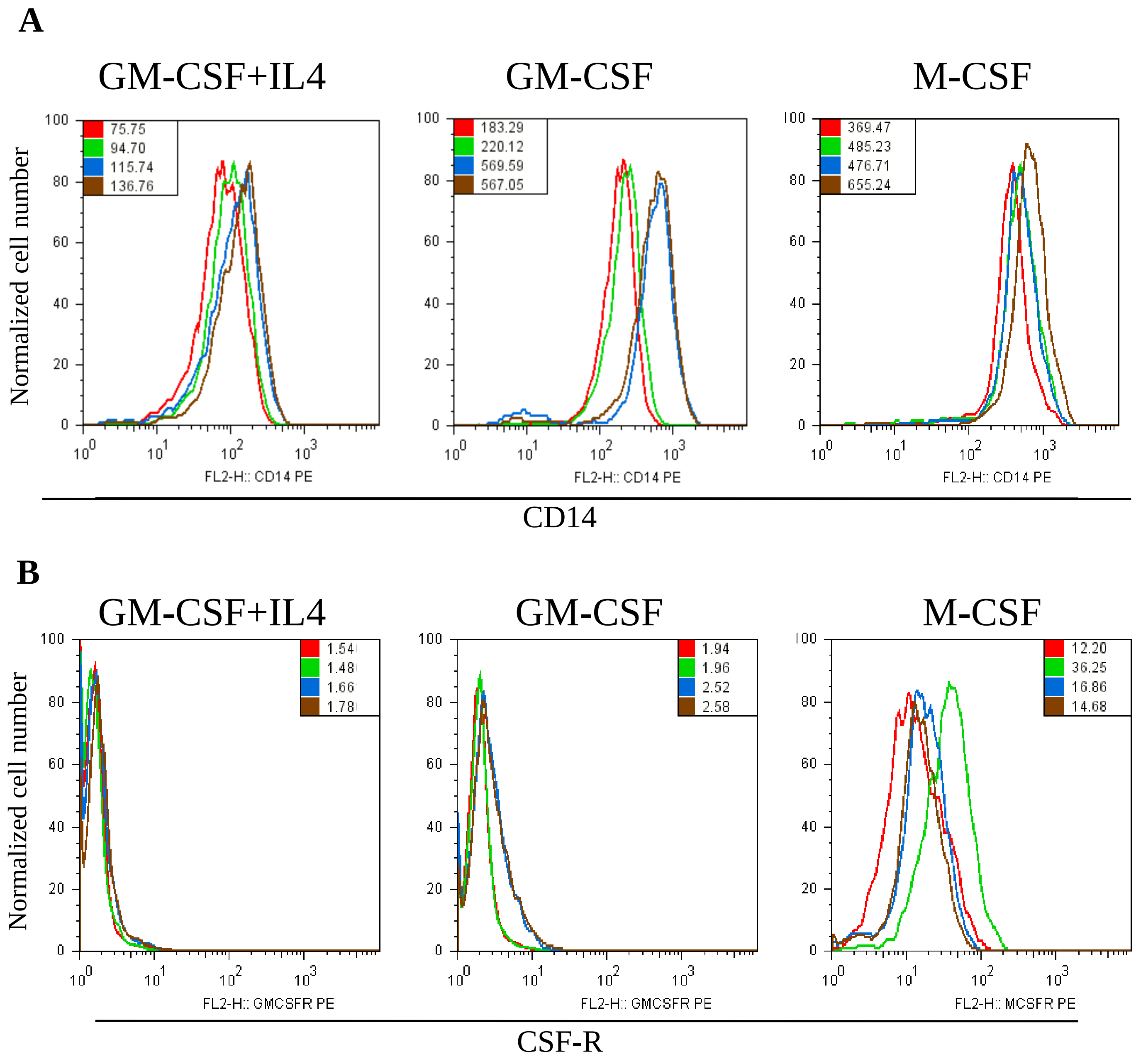

Supplementary Figure 3

A GM-CSF+IL4

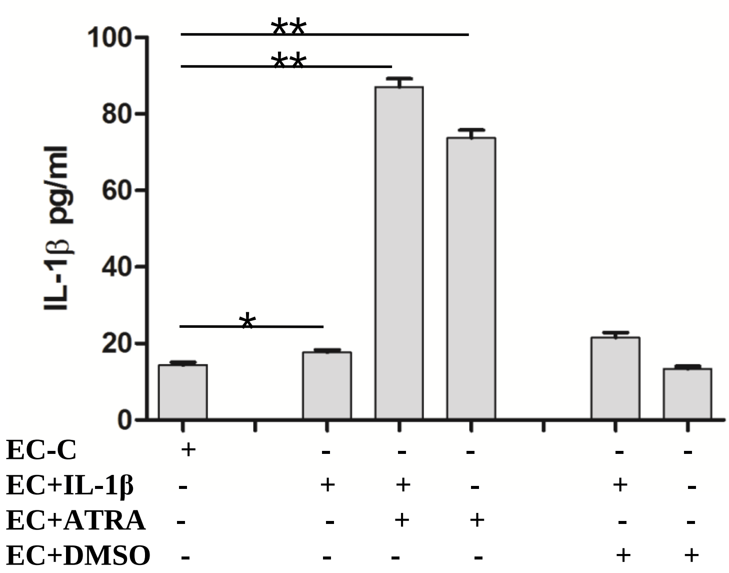

B GM-CSF

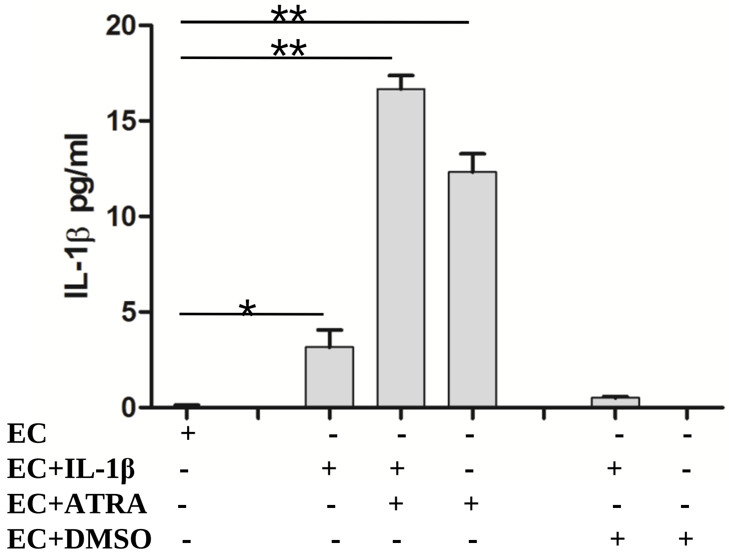

Supplementary Figure 4

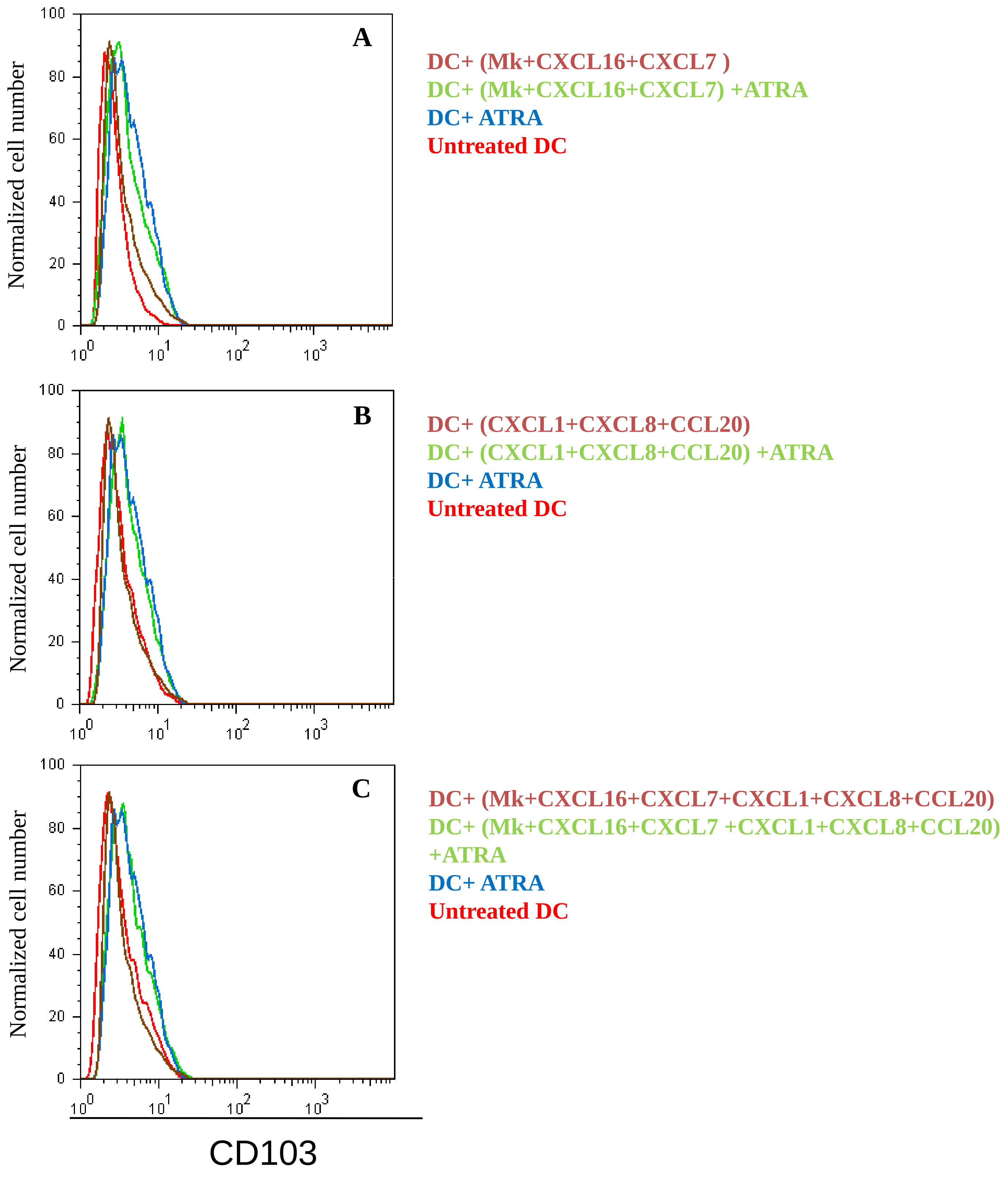

Supplementary Figure 5

[**GM-CSF+IL-4** differentiated] DC + **IL-1β** activated Caco2 spn + CD4$^+$T-cell

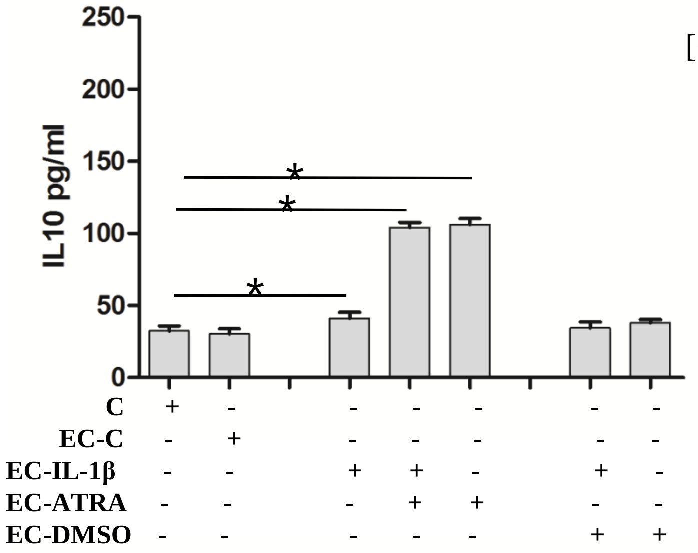

[**GM-CSF** differentiated] DC + **IL-1β** activated Caco2 spn + CD4$^+$T-cell

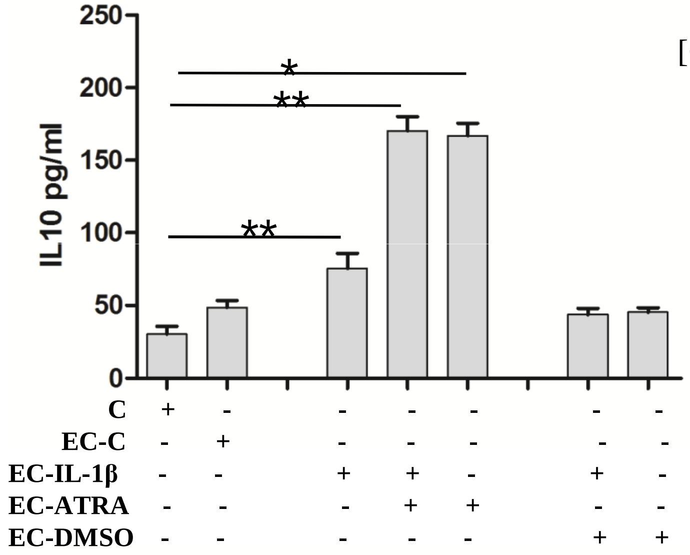

[**M-CSF** differentiated] Mf + **IL-1β** activated Caco2 spn + CD4$^+$T-cell

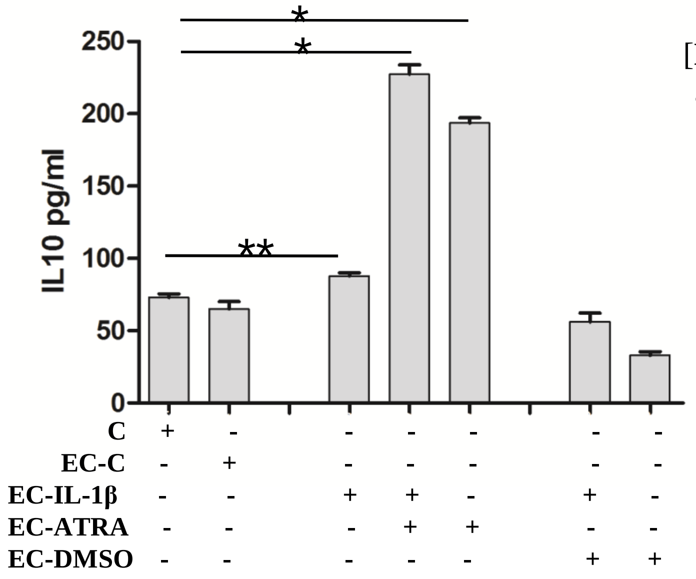

Supplementary Figure 3

Caco2 cells were treated with IL-1β in the presence of ATRA for 1hr. The culture medium of Caco2 was removed, replaced with fresh medium and the cells were cultured for an additional 5hrs. The supernatant was collected and added to monocytes differentiating in GM-CSF+IL4 or GM-CSF. The autocrine secretion of IL-1β induced by the activated Caco2 supernatant was measured by ELISA. The autocrine secretion of IL-1β by monocyte-derived cells was observed exclusively when the monocytes were generated in the presence of GM-CSF. Bar diagrams indicate mean±SD of 3 independent experiments $*p<0.05$ $**p<0.01$

Supplementary Figure 4

A – DC were treated with the mixture of chemokines Mk+CXCL16+CXCL7 in the presence and absence of ATRA. **B** – DC were exposed to the mixture of chemokines CXCL1+CXCL8+CCL20 in the presence and absence of ATRA. **C** – DC were exposed to the mixture of chemokines Mk+CXCL16+CXCL7+CXCL1+CXCL8+CCL20 in the presence and absence of ATRA. MFI of a typical measurement out of 3 independent experiments is shown.

Supplementary Figure 5

Secretion of IL-10 by $CD4^+$T-cells after co-culturing them with DC and Mf previously 'educated' by pretreatment with ATRA conditioned Caco2 supernatant. In the figure legends, C stands for unstimulated myeloid cells co-incubated with $CD4^+$ T cells, EC-C for monocyte-derived cells 'educated' by resting Caco2 supernatant followed by co-incubation with $CD4^+$ T cells, EC-IL-1β, EC-TNF-α, EC-ATRA, EC-DMSO correspond to cultures containing monocyte-derived cells 'educated' with the supernatants of CEC pretreated with IL-1β or

TNF-α in the presence or absence of ATRA, or with DMSO used as solvent control, followed by co-incubation with $CD4^+$ T cells. Bar diagrams indicate mean±SD of 3 independent experiments $^*p<0.05$ $^{**}p<0.01$
